# Abortion care process based on sexual and reproductive health and rights in Japan: A qualitative study

**DOI:** 10.1111/jjns.70010

**Published:** 2025-04-15

**Authors:** Yuka Sato, Akiko Haga, Chitaru Tokutake, Atsuko Samejima, Makoto Kanai, Satoko Nakagomi

**Affiliations:** ^1^ School of Health Sciences Shinshu University School of Medicine Matsumoto Nagano Japan

**Keywords:** abortion, grounded theory, quality of health care, reproductive health services, reproductive rights

## Abstract

**Aim:**

This study aimed to clarify the process of abortion care based on sexual and reproductive health and rights (SRHR) practiced by Japanese midwives.

**Methods:**

The participants were 44 midwives with experience in abortion care. Data were collected based on the framework developed by the World Health Organization's *Clinical Practice Handbook for Safe Abortion* and through detailed interview sessions. The modified grounded theory approach was used for data analysis.

**Results:**

The abortion care process was classified into five categories, 16 subcategories, and 49 concepts. The category “supporting women in making their own choices in life and leading their lives in the future” emerged as a foundational attitude toward abortion care. Midwives accompany pregnant women in the decision‐making process with a neutral standpoint. They perform procedures to ensure that women experience comfort and safety throughout the abortion process. After a medical procedure, midwives attend to issues associated with SRHR, such as contraception and future pregnancies.

**Conclusions:**

Abortion care based on SRHR is a care practice that respects women's bodily autonomy and supports women in a non‐judgmental manner. Abortion care also aims to provide women with safe and comfortable medical treatment, thereby supporting them in progressing with hope to their post‐abortion lives. The study results will help midwives reaffirm the significance of abortion care, improve care quality in clinical settings, and contribute to the advocacy of women's SRHR.

## 1 INTRODUCTION

Abortion care is essential for protecting women's sexual and reproductive health and rights (SRHR). Access to sexual and reproductive health services is critical for progress toward universal health coverage (World Health Organization [WHO], 2023a). The coronavirus disease 2019 (COVID‐19) pandemic considerably limited access to sexual and reproductive health services (Senderowicz & Higgins, 2020). In response to the situation, the WHO added comprehensive abortion care to the list of essential health services in 2020 (WHO, 2020) and published a new abortion care guideline in 2022 (WHO, 2022). In the guideline, the quality of abortion care is defined by six dimensions: effective, efficient, accessible, acceptable/patient‐centered, equitable, and safe. The guideline further describes the four core components of an enabling environment for abortion care: respect for human rights, a supportive framework of law and policy, the availability and accessibility of information, and supportive health systems (WHO, 2022).

Advances that ensure access to quality abortion care and realize SRHR have been made worldwide. First, from a legal perspective, South Korea decriminalized abortion in January 2021 (International Planned Parenthood Federation, 2021), a significant step toward social change and SRHR promotion. Second, with regard to methods, abortion medicines have been approved in more than 100 countries as of May 2024 (Gynuity Health Projects, 2024). Medical abortion, a standard abortion procedure in the United States and Europe, represented 63% (Guttmacher Institute, 2024) and 96% (Socialstyrelsen, 2024) of all abortions in 2023 in the United States and Sweden, respectively. In addition, the demand for medical abortion through telemedicine and self‐managed medical abortion has increased in several countries since the COVID‐19 pandemic (Aiken, Starling, et al., 2021; Boydell et al., 2021; Kaller et al., 2021). Self‐managed medical abortion is a safe and effective process that protects a woman's privacy and dignity (Aiken et al., 2022; Aiken, Lohr, et al., 2021). The WHO lists abortion medicines as essential medications that should be widely available to everyone (WHO, 2023b). For this reason, medical abortions are covered by public health insurance in many countries.

In contrast, Japan faces many challenges in ensuring access to quality abortion care. First, Japanese law stigmatizes abortion and does not support women's reproductive autonomy. In Japan, induced abortion is criminalized under the Penal Code (Ministry of Justice, 2017). Although the Maternal Health Act (Ministry of Justice, 2013) legalizes abortion under certain conditions, only designated obstetricians and gynecologists are allowed to provide abortions, and the spouse's consent is required. Next, medical abortion has not been established as a treatment option in Japan. Curettage is a common procedure in early pregnancy abortion (Nakamura et al., 2021); however, the WHO does not recommend the procedure given the associated complications (WHO, 2022). Abortion medicines were approved in April 2023 in Japan. In the 6 months following approval, 435 of a total of 36,007 abortions were medical abortions (MHLW, 2024b). A nationwide survey of 2000 women in Japan indicated that awareness of abortion medicines was only 29% (Linepharma, 2024). Finally, the cost of abortion is problematic. Induced abortion is considered a selective treatment and is not covered by public health insurance in Japan. Instead, the cost of abortion treatment is determined by each medical institution (Kaneda, 2023). In particular, young people and those with financial difficulties may be unable to access abortion given the high cost.

Midwives are indispensable in the provision of quality abortion care. The International Confederation of Midwives (2014) states that a midwife “acknowledges women as persons with human rights and seeks justice for all people and equity in access to health care.” Furthermore, abortion care is an essential competency of midwifery practice because midwives must provide care to support women's health and rights.

Japanese midwives provide counseling, procedure assistance, and health checks in abortion care. However, the Core Competencies for Japanese Midwives (Japan Midwives Association, 2021) does not include a specific description of abortion care practice. Moreover, Mizuno (2014) demonstrated that abortion care education in Japanese nursing and midwifery programs is insufficient. Another study indicated that being involved in both abortion and childbirth care creates confusion and raises ethical questions among Japanese midwives (Mizuno, 2011). Many midwives and nurses feel aversion toward women who have abortions and provide care reluctantly (Katsumata, 2018; Mizuno et al., 2025; Shimoyama & Tsukamoto, 2021). Abortion care performed under such circumstances cannot guarantee women's SRHR.

With the approval of abortion medicines, abortion care in Japan must catch up with the global standard. As a first step, an abortion care method that protects women's SRHR, can be applied to the contemporary Japanese legal and healthcare systems, and can be performed by midwives in the scope of their current duties must be established. This study aims to clarify the abortion care process based on SRHR that is suitable for Japan and conforms to midwives' perspectives and experiences.

### 1.1 Definition of abortion and abortion care in Japan

Abortion includes not only induced abortions but also miscarriages. Induced abortion is performed only for pregnancies of less than 22 weeks when the woman's physical health is damaged for “bodily or economic reasons” or when the pregnancy results from violent acts or coercion (Ministry of Justice, 2013). Early‐stage abortion (less than 12 weeks of pregnancy) is distinguished from middle‐stage abortion (more than 12 weeks and less than 22 weeks of pregnancy). In this study, abortion was defined as induced abortion within 22 weeks of pregnancy.

Notably, abortion is performed in six out of 10 cases in unintended pregnancies and in three out of 10 cases of all pregnancies worldwide (WHO, 2022). According to the Ministry of Health, Labour and Welfare (MHLW, 2024a), the number of abortions in fiscal year 2023 was 126,734, whereas the number of births was 727,288 in the same year. The MHLW report further indicates that more than half of all abortion cases were those in women in their teens or 20s. Most of these abortions were performed in the early stages of pregnancy for reasons related to patient health.

Abortion care in this study included the provision by midwives of consultation, counseling, and information about abortion procedures and post‐abortion follow‐up care.

## 2 METHODS

### 2.1 Study design

The modified grounded theory approach (M‐GTA; Kinoshita, 2020), a qualitative study method, was employed in this study. According to Kinoshita (2020), the M‐GTA study method generates an original theory that explains the interactive process from analysis that is grounded on data. The theory emphasizes practical applications in clinical settings similar to the sites where the data were collected. M‐GTA was developed by adopting the theoretical and contextual properties of the grounded theory approach (Glaser & Strauss, 1967). The theoretical framework of the grounded theory approach is derived from insights into symbolic interactionism (Holloway & Wheeler, 1996). The purpose of this study is to generate a theoretical basis for Japanese midwifery abortion care as practiced through the interaction between midwives and women. Therefore, M‐GTA is the most appropriate approach for this study.

### 2.2 Participant recruitment and selection criteria

The study participants were midwives who recognized the significance of women's SRHR. The initial recruitment target was midwives who belonged to an organization engaged in sexuality education activities based on scientific evidence (i.e., a sexuality education organization) and five medical institutions. These midwives regularly advocate for the importance of abortion care based on SRHR through their clinical practice, sexuality education, and international healthcare activities.

The selection criteria were (1) midwives who belonged to a sexuality education organization and had experience in abortion care, (2) those who were currently engaged in more than one abortion care practice case monthly, or (3) those who had over 1 year of experience in engaging in more than one abortion care case monthly. The participants who satisfied any of the above criteria were recruited using snowball sampling. The participants were also selected using theoretical sampling. Abortions differ by women's backgrounds and reasons for abortion, the number of weeks of pregnancy, and the types of facilities where the procedure is conducted. Therefore, sampling midwives with varying levels of abortion care experience was necessary.

The participants confirmed their willingness to collaborate in the study by sending an e‐mail message to the researcher. After obtaining consent to participate in the study, the interview date was set according to the participants' requests.

### 2.3 Data collection

**FIGURE 1:**
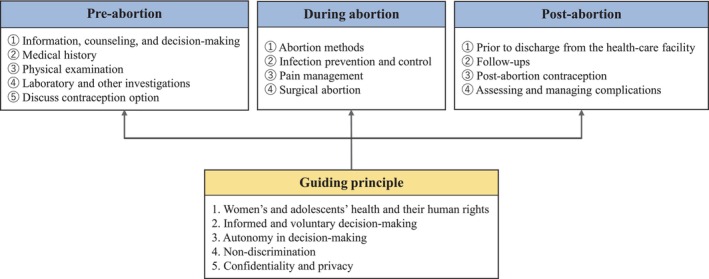
Interview framework to confirm details of specific abortion care practice by midwives. Developed based on *Clinical Practice Handbook for Safe Abortion* (WHO, 2014). During the interviews, we asked the participants about their perspectives and considerations regarding the care they provide to women before, during, and after abortion. In addition, we focused on how guiding principles were included.

Data were collected by the first author in semi‐structured interviews using a web‐meeting tool (Zoom) from May to August 2022. Kinoshita (2020) asserted that qualitative data represent complicated and diverse human experiences by providing richly detailed context. Online interviews were used because midwives across Japan were included in the study and the method ensured COVID‐19 infection control. Information on the participants' characteristics was collected using a questionnaire. Before the interview sessions, the participants were asked about their perceptions and experiences regarding abortion care to ensure the credibility of their narratives regarding abortion care. All of the participants demonstrated consistent commitment to abortion care to support SRHR. Next, the interview guide was developed (Figure 1). The guide referred to the care items described in the *Clinical Practice Handbook for Safe Abortion* (WHO, 2014) for pre‐abortion, during abortion, and post‐abortion care procedures. In the study, we used the interview guide to ask the midwives about the details of their abortion care practices. During theory generation based on constant comparative analysis, it was necessary to supplement the data obtained from two participants in the first interview to improve overall data interpretability; therefore, a second interview was conducted with each of the two participants. The first interview ranged from 37 to 98 min (mean: 61 min) per participant. The duration of the second interview, which was conducted with two specific participants, was 28 minutes for one participant and 60 minutes for the other (mean: 44 min). The interviews were recorded with the permission of the research participants and later transcribed verbatim.

### 2.4 Data analysis

Data were analyzed following M‐GTA procedures (Kinoshita, 2020). The theme of the analysis in this study was “the process of abortion care by midwives.” The focus of the analysis was “midwives who recognized the importance of abortion care from the SRHR perspective and had experience in abortion care.”

The analysis procedure was as follows: (i) the data were interpreted by focusing on the analytical themes, (ii) concepts with descriptive definitions were then generated, (iii) comparative analysis was repeated to examine similarities and counterexamples, (iv) intra‐concept relationships were investigated and organized into a relationship diagram after multiple concepts were generated, (v) categories were developed based on the relationships between the concepts, and (vi) concise summaries of these categories were documented and a result diagram was created. The theoretical saturation was considered attained when no new concepts or categories were developed in this field of study. In addition, researchers agreed that the phenomenon of the analyzed topic could be explained using the generated concepts and categories. Furthermore, to enhance the credibility and validity of the research process, the study was supervised in the setting of the research topic and data were analyzed by researchers who were familiar with qualitative research and experienced in medical matters and patients in abortion practice.

### 2.5 Ethical considerations

This study was approved by the Medical Ethics Committee of the authors' affiliated universities (Approval number: 5437). Written informed consent was obtained before conducting the interviews. The study's purpose was explained to each participant verbally and in writing, and the participants were assured that their data would be treated confidentially. Additionally, the participants were informed that their refusal to participate in the study would not disadvantage them.

## 3 RESULTS

Table 1 shows the characteristics of the 44 participants in this study.

**TABLE 1:** Participants characteristics.

	*N*	%
Age (*N* = 44)		
20s	6	13.6
30s	17	38.6
40s	14	31.8
50s and older	7	15.9
Current affiliations (*N* = 44)^a^		
Hospital	8	18.2
Clinic	23	52.3
Midwifery center (including only on‐site)	4	9.1
Health center, prefectural or municipal offices	1	2.3
Educational or research institute	1	2.3
Freelance (active without affiliation)	4	9.1
Others	9	20.5
Experience of abortion care education (*N* = 44)		
None	19	43.2
Only in coursework's of maternity nursing and midwifery training	2	4.5
Only after graduation	14	31.8
Both	9	20.5
Care experiences during abortion procedure (*n* = 43)^b^		
Patients less than 12 weeks of pregnancy		
None	2	4.7
Less than 5 cases	4	9.3
6–10 cases	0	0.0
11–20 cases	1	2.3
21–29 cases	4	9.3
30 cases and above	32	74.4
Patients more than 12 weeks of pregnancy		
None	2	4.7
Less than 5 cases	8	18.6
6–10 cases	3	7.0
11–20 cases	15	34.9
21–29 cases	3	7.0
30 cases and above	12	27.9

*Note*: Due to rounding, the total does not equal 100%.

^a^ Multiple response was allowed.

^b^ There was one among 44 participants who attended patients only before and after abortion procedures and did not have care experience during the procedure; therefore, the number of participants was *n* = 43.

**FIGURE 2:**
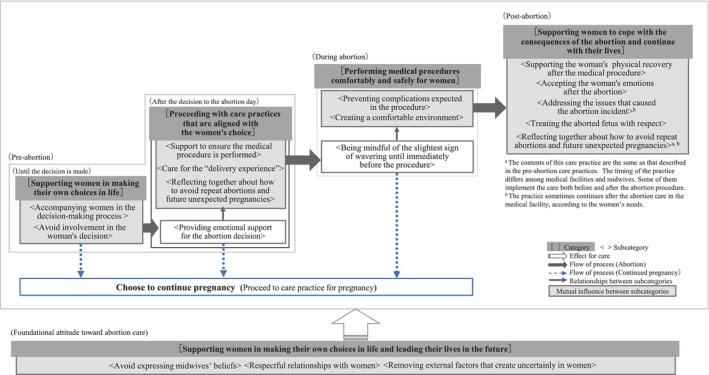
Abortion care process by midwives (result diagram).

Five categories, 16 subcategories, and 49 concepts were generated (Table 2a–d). The midwifery abortion care process consists of components described by five categories, and the interrelationships of the components are illustrated using 16 subcategories in Figure 2. Categories and concepts are indicated by the phrases in square brackets and quotation marks, respectively.

**TABLE 2:** (a) Foundational attitude toward abortion care. (b) Pre‐abortion care procedure. (c) Care during the abortion procedure. (d) Post‐abortion care procedure.

Category	Subcategory	Concept
(a)		
Supporting women in making their own choices in life and leading their lives in the future	Avoid expressing midwives' beliefs	Respecting women's wishes
		Supporting the woman's decision, regardless of whether it is childbirth or abortion
		Withholding midwives' feelings toward the fetus
	Respectful relationships with women	Giving the woman's privacy utmost consideration
		Determining the appropriate time and content for support based on a woman's behavior
		Emphasizing the importance of management of one's body
	Removing external factors that create uncertainly in women	Removal of visual and auditory stimuli
		Being prepared to listen to women at all times
		Choosing when to engage women in conversation by carefully observing women's emotional expressions
(b)		
Supporting women in making their own choices in life	Accompanying women in the decision‐making process	Providing information and search for solutions
		Telling the women that they do not have to suffer alone
		Providing time for women to think
	Avoid involvement in the woman's decision	Listening to women's true intention or feelings
Proceeding with care practices that are aligned with the women's choice	Providing emotional support for the abortion decision	Affirming the process that led to the abortion decision
		Accepting the woman's indecision that may stem from a sense of guilt
		Informing the woman that until the day of the abortion procedure, she can reverse her decision and continue her pregnancy
	Support to ensure the medical procedure is performed	Collecting information necessary for safe abortion practice
		Explaining the abortion and pain management procedure in easy‐to‐understand words
		Clarifying roles among the staff
		Referring women to medical facilities that are appropriate to their situations
		Maintaining connection
	Care for the “delivery experience”	Supporting preparation for ‘artificial stillbirth’
		Supporting women in preparing to welcome the stillborn baby
	Reflecting together about how to avoid repeat abortions and future unexpected pregnancies	Referring to the circumstances that led to the unexpected pregnancy/abortion
		Exploring the potential causes of repeat abortions
		Suggesting contraceptive methods suitable to the woman's situation
		Providing correct knowledge of contraceptive methods
		Supporting women in following their wishes regarding future pregnancies
(c)		
Performing medical procedures comfortably and safely for women	Being mindful of the slightest sign of wavering until immediately before the procedure	Do not rush to perform the procedure if the woman shows signs of wavering
	Preventing complications expected in the procedure	Proceeding with the established procedure
		Implementing infection control measures
		Preventing the woman's body from moving during the procedure
		Observing the woman's state to ensure the early detection of abnormalities
	Creating a comfortable environment	Do not make the woman wait
		Alleviating the woman's anxiety or tension
		Minimizing the pain the woman feels
		Attending the procedure in a manner that is not different from that of a normal childbirth (in cases of middle‐stage abortion)
(d)		
Supporting women to cope with the consequences of the abortion and continue with their lives	Supporting the woman's physical recovery after the medical procedure	Confirming the safe completion of the medical procedure
		Informing women about post‐abortion self‐management
		Confirming the women's daily lives
	Accepting the woman's emotions after the abortion	Informing women about the safe completion of the medical procedure and comfort them
		Listening to women's differing feelings about abortion
		Respecting the women's choice regarding abortion
		Coordinating schedule to ensure that the same staff can attend to the woman as much as possible
		Informing the women that they can rely on the medical facility anytime
	Addressing the issues that caused the abortion incident	Determining the problem specifically
	Treating the aborted fetus with respect	Attending to the aborted fetus as a precious ‘life’
		Preparing the visitation according to the women's wishes
		Supporting the women to keep the memory of the unborn fetus
	Reflecting together about how to avoid repeat abortions and future unexpected pregnancies^a^	Referring to the circumstances that led to the unexpected pregnancy/abortion
		Exploring the potential causes of repeat abortions
		Suggesting contraceptive methods suitable to the woman's situation
		Providing correct knowledge of contraceptive methods
		Supporting women in following their wishes regarding future pregnancies

^a^ The contents of this care practice are the same as that described in the pre‐abortion care practices. The timing of the practice differs among medical facilities and midwives. Some of them implement the care both before and after the abortion procedure.

### 3.1 Storyline

An overview of midwifery abortion care is shown below as a storyline.

The foundational attitude toward abortion care was [supporting women in making their own choices in life and leading their lives in the future]. The midwife respects the woman's choice and provides respectful care. The abortion care process starts with the decision‐making phase. Midwives provide women with the necessary information and neutrally accompany women in their decision‐making, thus [supporting women in making their own choices in life]. After women have made their decisions, midwives focus on [proceeding with care practices that are aligned with the women's choice]. Midwives prepare women for abortion by attending to women's feelings. By considering the women's feelings for their unborn children, midwives provide the same care that is given during childbirth, particularly in mid‐term abortions. Midwives carefully observe women's behavior until the moment of the medical procedure because they recognize that some women do not abandon hope for childbirth and childcare even after deciding to have an abortion. When midwives perceive that a woman is uncertain about her decision, they advise the woman to reconsider. As a result, some women reverse their decision and choose to continue the pregnancy. During the abortion procedure, midwives ensure that they are [performing medical procedures comfortably and safely for women]. After the abortion procedure, midwives focus on [supporting women to cope with the consequences of the abortion and continue with their lives], thus helping to promote women's physical recovery and the acceptance of their emotions. Furthermore, midwives coordinate collaboration among relevant organizations, provide information regarding contraception and future pregnancies, and support the women's fresh start.

### 3.2 Components of the midwifery abortion care process

Each category is a component of the abortion care process and is constructed from subcategories. The subcategories are described using the concepts in the following sections. Moreover, participants' quotations are included as supplementary explanations.

#### 3.2.1 Foundational attitude toward abortion care

##### Avoid expressing midwives' beliefs

Midwives ensure that they “respect women's wishes” and “support the woman's decision, regardless of whether it is childbirth or abortion” as crucial. Midwives sometimes feel sad or angry when reflecting that the woman's choice prevents the birth of the fetus. Midwives' ability to “withholding their feelings toward the fetus” helps a midwife focus on supporting the woman.

We do not hold prejudices or judge women who repeatedly receive abortion treatment. We support them by respecting their decision to have an abortion. (Case 8)

##### Respectful relationships with women

Midwives “give the woman's privacy utmost consideration” because they understand that abortion care can intrude on the woman's circumstances. Crucially, midwives “determine the appropriate time and content for support based on a woman's behavior” and not unilaterally impose information or care from the medical provider. Midwives also desire that women gain insights and awareness from the experiences of pregnancy and abortion, and they “emphasize the importance of management of one's body.”

##### Removing external factors that create uncertainty in women

Midwives must “remove visual and auditory stimuli” to avoid confusion in the woman. For example, midwives prepare an environment that is removed from the sounds of newborns or women in childbirth. Midwives always “prepare to listen to women at all times” and “choose when to engage women in conversation by carefully observing women's emotional expressions.” Thus, midwives constantly supervise women to provide care in the appropriate situation and at a suitable time.

For the woman who has decided to have an abortion after much emotional turmoil, we carefully ensure that she will not hear the sounds of newborns or meet other outpatients and thereby regret her choice. (Case 3)

#### 3.2.2 Pre‐abortion care procedure

##### Accompanying women in the decision‐making process

Midwives accompany women in their decision‐making process. They ask women why they are reluctant to continue their pregnancies, and then midwives neutrally “provide information and search for solutions” regarding childbirth and abortion. When women have difficulty deciding, midwives advise them to discuss the decision with their partners or families and “tell the women that they do not have to suffer alone.” After explaining the abortion method, and the possible window for abortion, midwives “provide time for women to think” based on the weeks of pregnancy.

Various other options including foster care or public financial assistance are available. It is a shame if a woman chooses to have an abortion without knowing about these options and regrets her decision later. (Case 19)

##### Avoid involvement in the woman's decision

Pregnancy, childbirth, and abortion are all experienced by a woman's body. Therefore, midwives suggest that women make their decisions their priority. The decision between childbirth and abortion may be influenced by circumstances or the opinions of others. Midwives refrain from stating their opinions on the matter; they only “listen to women's true intention or feelings” and respect that women decide for themselves.

##### Providing emotional support for the abortion decision

Midwives show respect to women who make the unavoidable decision to abort despite their worries and hesitation. First, midwives “affirm the process that led to the abortion decision.” Some women feel guilty about abortion. Midwives “accept the woman's indecision that may stem from a sense of guilt.” In addition, midwives consider that women may continue to show reluctance even after the decision to abort. After determining the date of the procedure, midwives “inform the woman that until the day of the abortion procedure, she can reverse her decision and continue her pregnancy.”

##### Support to ensure the medical procedure is performed

Midwives “collect information necessary for safe abortion practice” through interviews and physical examinations. To reduce women's fears and promote understanding, midwives “explain the abortion and pain management procedures in easy‐to‐understand words.” Midwives ensure that the abortion procedure progresses without delay by “clarifying roles among the staff” in advance.

Some women are unable to have the abortion procedure at the medical facility where they visit first given factors such as the number of weeks of pregnancy, complications, and medical history.

In such cases, midwives ensure that these women have access to abortion by “referring women to medical facilities that are appropriate to their situations.” If women are young (e.g., <18 years) and have limited support, midwives “maintain connection” to ensure that the abortion procedure is performed. The midwife remains involved to ensure that the woman does not lose access to medical care and support.

##### Care for the “delivery experience”

Although some women initially choose to give birth, they ultimately decide to terminate their pregnancies because of the unexpected development of the fetus or physical problems during pregnancy. Midwives attend to women's emotions to “support preparation for ‘artificial stillbirth’” and ensure that the abortion leads to a satisfactory birth experience. In addition, depending on the woman's wishes, midwives “support women in preparing to welcome the stillborn baby.”

If they choose a middle‐stage abortion, we ask them to tell us everything about their emotional conflicts, feelings, and the process that led to the decision. If they begin to feel like “doing something” for the child, we provide the care to make it happen. (Case 35)

##### Reflecting together about how to avoid repeat abortions and future unexpected pregnancies

First, midwives “refer to the circumstances that led to the unexpected pregnancy/abortion.” Specifically, midwives explore women's backgrounds and issues with their relationships with their partners (e.g., intimate partner violence). Women who have repeat abortions may experience problems such as sexual violence, economic difficulties, and developmental delays. Midwives “explore the potential causes of repeat abortions” and “suggest contraceptive methods suitable to the woman's situation.” Midwives provide detailed explanations of the contraceptive methods that the women can choose (i.e., “providing correct knowledge of contraceptive methods”). If necessary, midwives encourage the presence of the partner or parents.

Some women who decide to have an abortion express the desire to experience pregnancy and childbirth at a later date. Midwives “support women in following their wishes regarding future pregnancies.” This component of the abortion care process may be implemented after the abortion procedure. However, given the restrictions of the medical system (e.g., lack of time to talk to the patient after the abortion) and the physical burden on the woman after the abortion, the midwife provides this care before the abortion procedure.

If she mentions that she regrets the current pregnancy, we ask her about the contraceptive method she usually uses to suggest a new one for the next time. (Case 9)

#### 3.2.3 Care during the abortion procedure

##### Being mindful of the slightest sign of wavering until immediately before the procedure

The midwives observe the women's behavior until just before the abortion procedure and “do not rush to perform the procedure if the woman shows signs of wavering.” When midwives sense uncertainty in the women, they encourage the women to confront their feelings to ensure that they avoid regretting their decision later. This support causes some women to choose to continue their pregnancies.

Some women begin to shed tears when they enter the operating room. We sometimes advise on such occasions and tell them “to accept the procedure only when [they] feel 100% certain about it. It is OK to stop now and try again later.” In one case, laminaria were inserted in a woman for cervical dilation but later removed. (Case 17)

##### Preventing complications expected in the procedure

“Proceeding with the established procedure” ensures that the operation is performed safely and smoothly. “Implementing infection control measures,” such as applying standard precautions, using sterilized instruments, and administering antibiotics are essential. Midwives immobilize the woman's body and administer sedatives to “prevent the woman's body from moving during the procedure.” They also “observe the woman's state to ensure the early detection of abnormalities.”

##### Creating a comfortable environment

Creating a comfortable environment reduces the anxiety and tension that women feel during the medical procedure. To this end, midwives proceed with timely preparations and “do not make the woman wait.” Midwives touch the woman's body and talk to her to “alleviate the woman's anxiety or tension” and “minimize the pain the woman feels.” In addition, midwives collaborate with doctors to assess the woman's pain level and adjust the dosage of analgesics and sedatives.

The method of middle‐stage abortion is delivery. To ensure that the woman perceives the event as a birth experience, midwives “attend the procedure in a manner that is not different from that of a normal childbirth.”

By talking to her, she can feel relaxed, and I want her to know that she deserves good care. It is much better than leaving it to the doctors and doing nothing. (Case 13)

#### 3.2.4 Post‐abortion care procedure

##### Supporting the woman's physical recovery after the medical procedure

After the medical procedure is finalized, midwives “confirm the safe completion of the medical procedure.” Before women leave the medical facility, midwives “inform them about post‐abortion self‐management.” For example, midwives emphasize the importance of taking care of oneself daily, having emergency contacts, and ensuring timely medical examinations.

Midwives “confirm the women's daily lives” during follow‐up examinations. Specifically, they confirm if the women can lead her daily life without physical effects from the medical procedure.

##### Accepting the woman's emotions after the abortion

Midwives “inform women about the safe completion of the medical procedure and comfort them.” Midwives empathize with women's relief when the procedure is completed. Midwives also understand that abortion means different things to different women. They “listen to women's differing feelings about abortion” without denying them their feelings of liberation, grief, guilt, or hope. Midwives “respect the women's choice regarding abortion” because they understand that the decision to abort—which is associated with hesitation and conflict in some women—is necessary. If midwives assess that a woman requires continuous support after the abortion, they “coordinate schedules to ensure that the same staff can attend to the woman as much as possible.” This approach builds women's trust and confidence in midwives. Moreover, recognizing the psychological effects of the abortion procedure on women, midwives “inform the women that they can rely on the medical facility anytime.”

There is a woman who is carrying guilt for having “killed” her unborn child. I listen to her with a sincere feeling of respect, because she has made an important choice in her life. (Case 15)

There was a teenager who talked about her dreams for the future after her abortion procedure. Although ours was a one‐day interaction, I felt it was important to make time to listen to her. (Case 27)

##### Addressing the issues that caused the abortion incident

Midwives “specifically determine the problem” by referring to the circumstances that led to the abortion.

Midwives cooperate with relevant organizations such as sexual assault support services, police, schools, mental health departments, genetic counselors, grief care groups, and peer support groups to address the women's underlying issues.

##### Treating the aborted fetus with respect

Midwives “attend to the aborted fetus as a precious ‘life’” in the same way they attend to the fetus in miscarriage or stillbirth.

Before the abortion, midwives determine whether the women wish to meet the fetus or make memories. They again ask the women what they want post‐abortion, a period when some women change their minds. Midwives “prepare the visitation according to the women's wishes” and “support the women to keep the memory of the unborn fetus.” Midwives understand that this care should not be imposed unilaterally and that the women's wishes are the top priority.

We never specifically ask the patient if she wants to meet the aborted fetus because we do not want her to feel guilty when she chooses not to. We only ask her what she wants to do and wait for her answer to avoid forcing her into unwanted actions. (Case 3)

## 4 DISCUSSION

In this study, we used M‐GTA to clarify the abortion care process of Japanese midwives based on SRHR. The characteristics of abortion care by Japanese midwives based on SRHR, the implications for nursing practice, limitations, and further study topics are discussed in the following sections.

### 4.1 Characteristics of abortion care by Japanese midwives based on SRHR

#### 4.1.1 Supporting women's life decisions with a non‐judgmental attitude

The core support in abortion care based on SRHR is to support women's decision making in a non‐judgmental manner.

Japanese midwives often experience conflict between professional and personal values or have ethical issues related to the life of an aborted fetus (Mizuno, 2011; Mizuno et al., 2025). Similar to participants in previous studies, some of our study participants expressed having an ethical dilemma or personal feelings regarding the reasons for abortion or the aborted fetus. However, midwives controlled their emotions and never judged women's decisions. A possible factor that enabled this attitude was midwives' acceptance of the choices women made for their lives. The participants have met women with diverse backgrounds, felt empathy for them, and experienced the emotional turmoil that the women underwent. Eventually, the midwives realized that only the woman herself could lead her life. This realization has conferred midwives with a non‐judgmental attitude in their support of women's lives.

Decision‐making regarding abortion for Japanese women is not simple. First, Japanese law requires the mandatory consent of the spouse. As a result, a woman who is unable to obtain her spouse's consent is forced to proceed with an unwanted pregnancy, contrary to her preference. Many Japanese obstetrics and gynecology medical practitioners often already view a pregnant woman as a mother, and consider those who seek abortion as evil (Atsuta, 2017). Conversely, Japanese midwives and social workers often negatively view the decision to continue a pregnancy of single mothers or women with insufficient parenting capacity due to poverty (Ishiharada, 2017). Several studies have argued the importance of helping women who face pregnancy conflicts make relevant decisions proactively (Ueno et al., 2020) and the significance of evaluating whether women make their decisions regarding abortion care with utmost certainty (Gould et al., 2012). Similarly, our study results indicated the significance of decision‐making support that respects women's bodily autonomy. Midwives do not try to compel women to choose either childbirth or abortion; they attempt to ascertain the women's wishes and support them regardless of their choices. This care concept likely reflects the midwife's wish for the woman to make relevant decisions, as pregnancy has significant implications for her physical health and life plans.

#### 4.1.2 Conducting comfortable and safe medical procedures for women

Intra‐abortion treatment care involves providing women with comfort care in collaboration with the doctor to ensure the medical safety of the procedure and reduce women's pain and anxiety.

Although curettage, which the WHO does not recommend, is the abortion method most commonly employed in Japan (Nakamura et al., 2021), few medical incidents have been reported (Mizuno, 2015). Many participants' narratives supported that necessary infection control and complication prevention measures are appropriately implemented and precisely follow the WHO's recommendations (2022). Safe abortion treatment reduces women's physical risks and protects their future ability to become pregnant and give birth at the time they desire.

According to Makleff et al. (2019), women who undergo abortions have concerns about self‐stigma or being negatively judged by medical staff. As a participant's narrative that supports the concept “I want her to know that she deserves good care” indicates, the participants in this study provided comfort care to all women equally as a health care service they deserved. The study results provide the insight that comfort care is not simply a care practice to reduce pain or anxiety but part of comprehensive support that enhances women's dignity and self‐esteem.

#### 4.1.3 Supporting women to progress with hope in the life they choose

Abortion care based on SRHR is a care practice that supports women in overcoming their challenges and prepares them for the realization of the life they desire.

The emotional status of the woman post‐abortion includes a sense of regret, loss, guilt, shame, and relief (Broen et al., 2004; Major et al., 2000). The participants in this study exhibited a receptive attitude without denying the emotional expressions of the women to which they attended. This receptive attitude of midwives indicates their respect for the women who proactively make one of the most challenging choices of their lives and midwives' wish to provide hope to these women, who are preparing to take a new step forward. Midwives must recognize the importance of not stigmatizing women.

Japanese nurses provide “non‐intrusive care” in cases of early‐pregnancy abortion and refrain from actively engaging in communication with the women to avoid making them uncomfortable (Katsumata, 2018). Although such passive care practices can protect women's privacy, they may be insufficient in providing women with the necessary support. Reasons for seeking abortion include unwanted pregnancy, health issues, financial difficulties, and domestic violence from intimate partners (Biggs et al., 2013; Chae et al., 2017). The study participants first attempted to understand issues that underlay women's pregnancy and abortion, coordinated collaboration with relevant organizations or experts, and provided women with information regarding contraception and future pregnancies. Therefore, proper abortion care aims to enhance women's ability to manage their bodies and provides an excellent opportunity to support these women's self‐realization.

### 4.2 Implications for nursing practice

To the best of our knowledge, this is the first study to elucidate the abortion care process followed by midwives in Japan who respect SRHR. If the theory generated in this study is applied widely across abortion care sites in Japan, midwives would be expected to play a central role in supporting SRHR. The results of this study are also expected to foster support of quality care practice based on SRHR in future midwives by introducing them to basic and postgraduate education.

Abortion care in Japan has long been discussed from the perspective of grief care that focuses on mid‐term abortion and the necessity of care for women who experience distress and grief from having to undergo abortion for reasons such as fetal disorder (Cancerscan, 2022). In contrast, abortion care based on SRHR perspectives has progressed poorly. Midwife‐led abortion care, related education systems, and the commitment by midwives to support access to SRHR have been established in many countries (Fullerton et al., 2018; Mainey et al., 2020; McNamara et al., 2023). In contrast, providing abortion is not included in the scope of midwives' duties in Japan; midwives work under doctors' supervision. In addition, the current insufficient level of abortion practice training in midwifery education in Japan (Mizuno, 2014) has a grave impact. The results of this study emphasized the significance of the SRHR perspective in abortion care in Japan and demonstrated the potential of midwives' contribution to improving access to quality abortion care.

### 4.3 Limitations and further study

This study has two limitations. First, the care practice observed in this study lacks the opinions of the women who receive abortion care. Second, previous studies have argued that many midwives in Japan exhibit negative attitudes toward abortion care practice. Currently, the assessment of the quality of abortion care is based on each individual's perceptions or value judgments. Thus, the results of this study cannot be considered generally applicable to all midwives in Japan.

As a further investigation, relevant data from the perspectives of women who undergo abortion should be accumulated to guide patient‐oriented abortion care based on SRHR. Study results can also guide the development of appropriate education systems and training environments. Spreading SRHR education and providing midwives with opportunities to reflect on value creation are steps toward the realization of quality abortion care. Moreover, to the best of our knowledge, no studies have elucidated Japanese midwives' perceptions of or roles in medical abortion. Further investigation is necessary in this regard, especially concerning how midwives in Japan who, unlike their counterparts in other nations, are not allowed to prescribe abortion medicines—can contribute to supporting medical abortion practices.

## 5 CONCLUSIONS

The abortion care process based on SRHR in Japan consists of four phases with a total of five categories, 16 subcategories, and 49 concepts extracted.

In Japan, midwives have a limited role in abortion care under the Maternal Health Act. Despite this limitation, Japanese midwives do their utmost to protect women's SRHR by supporting women's decisions in a non‐judgmental manner and respecting women's bodily autonomy. Additionally, midwives support women in progressing with hope to their post‐abortion lives by providing them with comfortable and safe medical treatment.

The results of this study can help midwives reaffirm the significance of abortion care, improve care quality in clinical settings, and contribute to advocating women's SRHR. In addition, the results of this study allow midwives to contribute to medical abortion practice to improve the quality of abortion care in Japan toward the global standard.

## AUTHOR CONTRIBUTIONS

Study design: Yuka Sato, Akiko Haga, Chitaru Tokutake, Atsuko Samejima, Makoto Kanai, and Satoko Nakagomi. Data collection: Yuka Sato. Data analysis: Yuka Sato, Akiko Haga, Chitaru Tokutake, Atsuko Samejima, Makoto Kanai, and Satoko Nakagomi. Manuscript writing: Yuka Sato and Satoko Nakagomi. All authors have approved the final manuscript.

## CONFLICT OF INTEREST STATEMENT

The authors declare that there is no conflict of interest.
